# Preparation of a Multifunctional Gel for Fire Prevention and Extinguishing Based on Polyvinyl Alcohol/Polyethyleneimine/Polyaluminum Chloride

**DOI:** 10.3390/polym18091017

**Published:** 2026-04-23

**Authors:** Jianguo Wang, Binyuan Gao, Yueyang Zhou

**Affiliations:** College of Safety Science and Engineering, Xi’an University of Science and Technology, Xi’an 710054, China; 13834846998@163.com (B.G.); 13834846998@139.com (Y.Z.)

**Keywords:** mine fire safety, gel, flame-retardant mechanism, functional groups, controllable gelation

## Abstract

A ternary gel composed of polyvinyl alcohol (PVA), polyethyleneimine (PEI), and polyaluminum chloride (PAC) was prepared to address the limited controllability of gelation and the insufficient high-temperature resistance to re-ignition observed in existing mine fire prevention and extinguishing gels. Based on an orthogonal experimental design, the optimal formulation was identified as 14% PVA, 7% PEI, and 5.5% PAC (by mass), achieving a gelation time of 8.2 min. Microscopic characterization revealed that the gel forms a dense, interconnected three-dimensional network structure capable of effectively encapsulating the coal particles. Fourier transform infrared spectroscopy (FTIR) analysis showed that gel treatment resulted in a 29.8% reduction in the peak area of free hydroxyl groups. Thermogravimetric–differential scanning calorimetry (TG-DSC) analysis indicated that the gel increased the ignition temperature by 33.27 °C and shifted the maximum exothermic peak temperature by 13.28 °C. Fire suppression experiments demonstrate that the gel could continuously lower the temperature of high-temperature coal without re-ignition, demonstrating significantly superior performance compared to traditional sodium silicate gel. This gel achieves highly efficient fire prevention and suppression through the cooperative effects of water retention, oxygen barriers, and chemical passivation, providing a new material for the prevention and control of spontaneous coal combustion in deep mines.

## 1. Introduction

Coal has long been dominant in China’s energy structure, and its status as a primary energy source is expected to remain so in the foreseeable future. It serves as a critical pillar for ensuring national energy security and economic stability [1,2]. During coal mining, more than 90% of mine fires are attributed to the spontaneous combustion of coal. With increasing mining depth and intensity, the mined-out area expands and air leakage intensifies, leading to a higher risk of spontaneous combustion [3,4]. Therefore, enhancing the prevention and control of spontaneous combustion of coal and reducing mine fire accidents are of great significance for ensuring personnel safety and the sustainable utilization of coal resources. Given the complex combustion environment of underground coal fires, and in the context of carbon neutrality goals, the development of multifunctional gel materials with excellent fire prevention and extinguishing performance is expected to achieve cooperative effects in preventing spontaneous combustion and reducing greenhouse gas emissions and is of significant research importance for coal mine safety and green mining [5,6,7]. Gel-based fire prevention and extinguishing technology integrates multiple functions, including sealing leaks, cooling, inhibition, heat absorption, and moisture retention [8,9].

By adjusting parameters such as pH and ionic strength, a novel self-assembled gel was developed. This material allows the modulation of pore structure and surface charge characteristics through the control of electrostatic interactions, thereby exhibiting high selectivity in the adsorption and separation of N_2_/CO_2_ [10]. In the field of polymer-based gels, spherical polyvinyl alcohol-based polymer microgels have been successfully prepared [11]. Additionally, materials combining thermal responsiveness with efficient fire prevention and extinguishing performance have attracted attention. Examples include a novel thermosensitive hydrogel based on hydroxypropyl methylcellulose and sodium alginate [12], as well as a self-healing carboxymethyl cellulose-based amphiphilic polymer hydrogel fabricated via ionic crosslinking between modified carboxymethyl cellulose and aluminum citrate [13]. Regarding structural reinforcement and stability, studies on polymer foam gel materials have shown that the gel skeleton maintains the structural integrity of the foam system, contributing to improved thermal capacity and stability [14]. Other work has focused on composite materials consisting of compound foaming agents and superabsorbent gels, along with a systematic analysis of their flame-retardant mechanisms [15]. Introducing environmentally friendly phosphonate salts into a chitosan/hydroxypropyl methylcellulose system was found to promote the formation of thermosensitive hydrogels, thereby enhancing both the structural strength and fire-extinguishing performance of the material [16]. Further research has explored the preparation and injection methods of various gel materials, comparing from a theoretical perspective the fire-suppression mechanisms and characteristics of different thickening colloids in coal mine applications [17]. In the field of gel foams, one study prepared gel foams by combining hydrophilic silica nanoparticles, xanthan gum, a fluorocarbon surfactant (FS-50), and a hydrocarbon surfactant (APG0810) [18]. The addition of nanoparticles and/or xanthan gum was observed to reduce the foaming capacity of the FS-50/APG0810 system and influence the foam drainage behavior. Additionally, a new gel system was developed using low-methoxy pectin, calcium bentonite, sodium bentonite (Na-Bt), and water [19]. The results indicated that the material exhibited excellent oxygen barrier properties, strong adhesion, high thermal stability, and significant inhibitory effects on coal combustion. Overall, gel-based fire prevention and extinguishing technologies have advanced considerably. However, with increasing mining depth and intensity, the underground environment has become more complex, and the spontaneous combustion of coal is influenced by multiple coupled factors. Existing technologies still exhibit limitations such as high costs, limited effectiveness, and insufficient environmental adaptability [20,21,22,23]. Therefore, the development of multifunctional gel materials with superior fire prevention and extinguishing performance is of great importance [24,25,26,27,28]. Such materials can effectively suppress spontaneous coal combustion, reduce the risk of mine fires, and ensure underground safety while providing both safety and environmental advantages. They also offer a new technical pathway for the cross-disciplinary application of gel materials in mine safety and low-carbon development.

However, traditional inorganic gels often suffer from inherent drawbacks, such as high brittleness and a tendency toward dehydration and cracking [29]. To address these limitations, researchers have introduced organic polymers, such as polyvinyl alcohol (PVA), for modification. Owing to its excellent water solubility, film-forming ability, and abundant hydroxyl groups, PVA can significantly enhance the toughness and water retention capacity of gels, and it has been widely used in the preparation of composite gel systems for mining applications [30]. To further improve the structural stability and functional performance of gel networks, the incorporation of a second reactive component is essential. Polyethyleneimine (PEI), a cationic polymer rich in amine groups, exhibits high reactivity and relatively low toxicity, making it a promising candidate for network modification [31,32,33,34]. In addition, polyaluminum chloride (PAC), as a low-cost and readily available inorganic crosslinking agent, can release Al^3+^ ions that rapidly coordinate with the hydroxyl groups of PVA to form a crosslinked network structure. Despite these advances, systematic investigations into the cooperative integration of PVA as a structural backbone, PEI as a network-enhancing agent, and PAC as a rapid crosslinker for constructing ternary gel systems tailored for mine fire prevention and extinguishing remain limited. Therefore, developing a ternary gel system that integrates these functionalities is of significant importance for achieving controllable gelation, enhanced thermal stability, and improved fire prevention performance.

Consequently, the present work combines theoretical analysis with a comparative experimental design to develop a multifunctional gel for fire prevention and extinguishing. By optimizing the formulation parameters, the gel exhibits appropriate gelation time, viscosity, and mechanical strength to meet the operational requirements of mining conditions. In addition, it demonstrates excellent thermal stability, enabling long-term coverage of coal surfaces and thereby achieving effective fire prevention, flame suppression, and fire extinguishing. The gel is characterized in terms of its macroscopic physicochemical properties, microstructure, and chemical composition using scanning electron microscopy (SEM), Fourier transform infrared spectroscopy (FTIR), and thermogravimetric–differential scanning calorimetry (TG–DSC), along with simulated mine experiments. Furthermore, its inhibitory effect on the spontaneous combustion of coal is comprehensively evaluated. The practical application performance is further verified through fire-extinguishing experiments.

## 2. Materials and Methods

### 2.1. Experimental Materials and Instruments

Polyvinyl alcohol (PVA; degree of hydrolysis ≥ 95%, Shanghai Meilin Biochemical Technology Co., Ltd., Shanghai, China), polyethyleneimine (PEI; 99%, Shanghai Meilin Biochemical Technology Co., Ltd., Shanghai, China), and polyaluminum chloride (PAC; 99%, Shanghai Yien Chemical Technology Co., Ltd., Shanghai, China) were used as received. Deionized water was used throughout the experiments.

The experimental equipment included an electronic balance (precision: 0.01 g), a laboratory electric stirrer (LC-ES-120), a rotational viscometer, and an electric thermostatic water bath (LC-WB-2). The electronic balance, laboratory electric stirrer, and water bath were all purchased from Lichen Technology Co., Ltd. (Shaoxing, Zhejiang, China).

### 2.2. Preparation Steps

A predetermined amount of PVA was added to deionized water in a beaker equipped with a mechanical stirrer. Upon initiating stirring, PVA was gradually and uniformly introduced into the solution and continuously stirred until complete dissolution was achieved, preventing the formation of aggregates.

PEI solutions with mass fractions of 7%, 8%, and 9% were prepared using deionized water. Each solution was thoroughly mixed and then stored at room temperature for subsequent use. Similarly, PAC solutions with mass fractions of 4.5%, 5.0%, and 5.5% were prepared using deionized water, ensuring complete dissolution before storage at room temperature.

The prepared PVA, PEI, and PAC solutions were mixed according to the proportions specified in the orthogonal experimental design. The solutions were rapidly combined in a beaker and immediately transferred to a thermostatic water bath maintained at 60 °C. Mechanical stirring was applied at 250 rpm and maintained until the system lost fluidity and exhibited typical gel characteristics. At this point, stirring was stopped, and the beaker was removed from the water bath, yielding the PVA/PEI/PAC ternary gel.

The entire mixing and gelation process was conducted under ambient conditions without additional pH adjustment, resulting in a final system pH of approximately 5–6.

### 2.3. Gel Base Performance Test

#### 2.3.1. Microstructure Characterization

Two sample systems were prepared. Sample 1 consisted of a gel formulated with 14% A, 7% B, and 5.5% C (10 g in total). Sample 2 was obtained by mixing the same gel (5 g) with coal particles (5 g, <0.3 mm). All samples were cured at room temperature for 24 h before characterization.

Scanning electron microscopy (SEM) observations revealed that the gel exhibited a uniform and continuous structure with a smooth surface, without obvious cracks or defects. The components formed a dense three-dimensional network, which contributed to the mechanical stability of the material.

Furthermore, the internal pore structure of the gel was uniformly distributed and well interconnected, which is beneficial for enhancing thermal stability and flame retardancy. These structural characteristics contribute to the improved performance of the gel in inhibiting the spontaneous combustion of coal.

#### 2.3.2. Gelation Time

The setting time of the gel was determined using a dripping method. The prepared gel solution was allowed to flow through a funnel, and the time required for complete dripping was recorded. The procedure was repeated until the dripping time increased by more than 50% compared to the previous measurement. This time point was defined as the setting time of the gel. Each formulation was tested in triplicate, and the average value was reported.

#### 2.3.3. Gel Viscosity

An NDJ-1 rotational viscometer was used to measure the time-dependent viscosity of the gel. To minimize structural disturbance, measurements were performed using a No. 3 rotor at 12 rpm, considering its high viscosity, which increases during crosslinking.

Four representative gel formulations selected from Section 2.2 were tested, namely 10% A + 8% B + 5.5% C, 12% A + 9% B + 5.5% C, 14% A + 7% B + 5.5% C, and 14% A + 8% B + 5.0% C. The viscosity evolution of each sample was recorded over a period of 0–20 min under ambient temperature conditions.

The effects of different component ratios on the viscosity development behavior of the gel were subsequently analyzed.

#### 2.3.4. Gel Strength

Gel strength was evaluated using the drop-rod method, which is simple to operate, cost-effective, and provides reasonable measurement accuracy. In this method, a glass rod is vertically positioned at a fixed height above the gel sample. Upon release, the rod falls freely under gravity, and the penetration depth into the gel is measured as an indicator of gel strength.

A greater penetration depth corresponds to lower gel strength, whereas a smaller penetration depth indicates higher strength.

#### 2.3.5. Exudation Test

The leakage performance of the gel was evaluated to assess its ability to penetrate coal seam fractures. Spontaneous combustion of coal typically occurs in deeper regions of the coal body. Therefore, for effective fire suppression, the gel must be capable of penetrating into fractures, covering the heat source, and isolating oxygen, thereby inhibiting fire propagation. The leakage test was designed to evaluate the diffusion capacity and penetration behavior of the gel within a simulated coal seam and to determine whether it can infiltrate pores and fractures to form a uniform and stable fire prevention barrier.

A leakage test apparatus was constructed to simulate the conditions of a fractured coal seam. The device consisted of an acrylic column with a length of 60 cm, an outer diameter of 50 mm, and an inner diameter of 46 mm. The column was filled with 1000 g of coal particles with mixed size fractions, including 0–0.9 mm, 0.9–3 mm, 3–5 mm, 5–7 mm, and 7–10 mm (200 g for each fraction), resulting in a packing height of approximately 45 cm. The bottom of the column was sealed with a plastic film perforated with several small holes to allow the test material to drain. A beaker was placed beneath the column to collect the leaked material.

During the experiment, an amount of gel equivalent to 10% of the coal mass (100 g) was uniformly poured from the top of the column. After the leakage process was completed, the mass of the collected material in the beaker was measured. The leakage rate was then calculated accordingly.

(1) $$\;\eta =\frac{m_{1}}{m_{2}}\times 100\%$$

In the formula, *η* is the leakage rate of the tested material (%), *m_1_* is the mass of the material that has leaked (g), and *m_2_* is the initial mass of the tested material (g).

### 2.4. Gel Fire Prevention and Extinguishing Performance Test

#### 2.4.1. Infrared Spectroscopy Test

Potassium bromide (KBr) was dried at 105 °C for 3 h using a 101-type electrically heated forced-air drying oven. The samples obtained by vacuum freeze-drying (as described in Section 4) were mixed with KBr at a mass ratio of 1:100 and ground uniformly. The mixture was then pressed into pellets with a thickness of approximately 1 mm under a pressure of 8–10 MPa.

The prepared pellets were placed in the sample chamber of a Fourier transform infrared (FTIR) spectrometer to ensure adequate contact with the infrared beam. The spectral measurements were carried out over a wavenumber range of 400–4000 cm^−1^ with 32 scans per sample. The corresponding infrared spectra were subsequently recorded.

#### 2.4.2. Quality and Thermal Change Characterization Test

Coal samples treated with a 5% gel and raw coal were dried in an oven at 35 °C for 24 h. The dried samples were then ground and sieved through a 100-mesh sieve for subsequent analysis. Prior to testing, the temperature and sensitivity of the instrument were calibrated to ensure measurement accuracy.

Approximately 10 mg of each prepared sample was weighed and placed in a crucible. Thermogravimetric–differential scanning calorimetry (TG–DSC) analysis was conducted under an air atmosphere. The airflow rate was set to 50 mL/min; the temperature range was 30–800 °C, and the heating rate was 5 °C/min. After the experiment, the data were recorded and exported for analysis.

Upon completion of the experiment, the furnace was allowed to cool naturally to room temperature before shutting down the system in accordance with standard operating procedures.

#### 2.4.3. Fire-Extinguishing Performance Test Setup

During the experiment, solid alcohol was used to ignite the coal sample. Simultaneously, a blower supplied air through the ventilation holes into the experimental system to increase the coal temperature. When the coal temperature reached 800 °C, the air supply was stopped, and the test materials were introduced.

**Figure 1:**
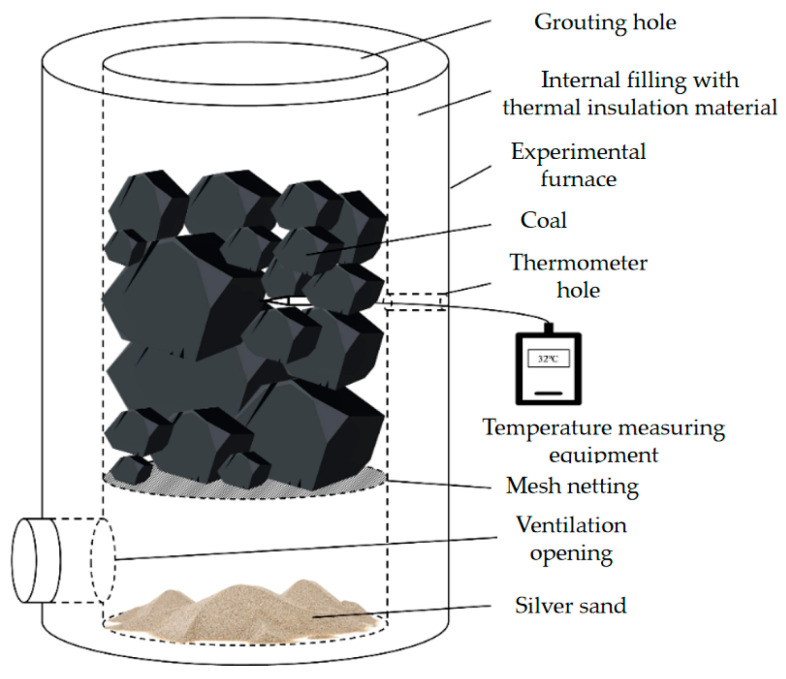
Gel fire-extinguishing experimental setup diagram.

The temperature was monitored using a TM-902C digital thermometer. Data were recorded at 10 s intervals, and the total test duration was 0–300 s. The fire extinguishing experimental setup is shown in Figure 1.

## 3. Results and Discussion

### 3.1. Analysis of Factors Affecting Gel Properties

An orthogonal experimental design with three factors (PVA, PEI, and PAC) and three levels was employed to evaluate the effects on gelation behavior.

Based on the proportions listed in Table 1, nine orthogonal experimental formulations were generated using SPSS software (2022), as shown in Table 1.

For PVA, concentrations below 10% are insufficient to form a continuous network, whereas concentrations above 14% result in excessively high viscosity, which is unfavorable for pumping and penetration. Therefore, three levels (10%, 12%, and 14%) were selected to systematically evaluate their effects on gelation behavior and material properties. For PEI, concentrations in the range of 7–9% can effectively regulate the gel structure without significantly affecting gelation kinetics; thus, three levels within this range were selected. For PAC, excessively high concentrations (>5.5%) lead to overly rapid reactions and very short gelation times, which are not conducive to deep fracture penetration. Accordingly, three levels (4.5%, 5.0%, and 5.5%) were chosen to achieve an optimal gelation window of 5–10 min.

Overall, the three factors and three levels constituted a 3-factor, 3-level orthogonal experimental design, enabling a systematic evaluation of factor effects with a reduced number of experimental runs.

The setting time of the gel was measured using a dripping method. The prepared gel solution was allowed to flow through a funnel, and the time required for complete dripping was recorded. This procedure was repeated until the gel dripping time increased by more than 50% compared with the previous measurement. This time point was defined as the setting time of the gel. Each formulation was tested in triplicate, and the average value was used to reduce experimental error.

According to the orthogonal experimental design, nine groups of gel tests were conducted, and the corresponding setting times were obtained. The orthogonal experimental results are presented in Table 2. Variance analysis (ANOVA) was performed using SPSS software to investigate the effects of each factor at different levels on the experimental results. In addition, ANOVA was used to identify the primary and secondary influencing factors and to evaluate their statistical significance.

To evaluate reproducibility, each orthogonal formulation was independently prepared and tested three times in parallel. The gelation times reported in Table 2 represent the arithmetic mean of three independent measurements, and the standard deviation was used to describe data dispersion.

Based on an orthogonal experimental design, nine gel formulations were prepared, and their gelation times were measured. Variance analysis (ANOVA) was used to evaluate the significance of each factor. Statistical analysis was performed using SPSS, and a high model fitting degree was obtained (R^2^ = 0.987). The results indicated that PVA (F = 23.258, *p* < 0.05) and PAC (F = 49.000, *p* < 0.05) had significant effects on gelation time, whereas PEI showed no significant effect (*p* > 0.05). The gelation time was mainly governed by PVA and PAC.

Considering the requirements of coal mine fire prevention and control, the gelation time was controlled within 5–10 min to ensure effective penetration into coal fractures. Based on these results, four formulations were selected for subsequent performance testing and optimization. The significance analysis of the effects of CS, AM, and MBA contents on gel formation time is shown in Table 3.

In the analysis of variance (ANOVA), the degrees of freedom (dfs) for each factor are determined by the number of levels and calculated using df = k − 1, where k represents the number of levels. Since each factor in this experiment was set at three levels, the corresponding degrees of freedom for each factor is two.

Based on the ANOVA results, the coefficient of determination (R^2^) was 0.987, indicating a good fit between the model and the experimental data. PVA showed a significant effect (F = 23.258, *p* = 0.041 < 0.05), indicating the presence of a main effect. This suggests that PVA significantly influences gelation time. PEI showed no significant effect (F = 4.290, *p* = 0.189 > 0.05), indicating that its effect on gelation time is not statistically significant. PAC showed a significant effect (F = 49.000, *p* = 0.020 < 0.05), indicating a strong main effect on gelation time. Overall, the influence of the three factors on gelation time follows the order of PAC > PVA > PEI, with PEI having a relatively minor effect. The gelation time is mainly governed by PVA and PAC.

The analysis of factor effects on gelation time provides a basis for adjusting gelation behavior to meet the requirements of different mining conditions, thereby improving the applicability of the gel-based fire prevention material.

For practical application in underground coal mine fire prevention and control, the gelation time should be neither too fast nor too slow and should be controlled within 5–10 min. Based on nine orthogonal experiments, the optimal gel formulation was determined to be 14% A + 7% B + 5.5% C.

The gelation time of 8.2 min achieved with the optimal formulation (14% PVA, 7% PEI, 5.5% PAC) originates from the cooperative interactions among the three components, which collectively govern a well-defined kinetic process.

1. Gelation Kinetics

The gelation process can be divided into three distinct stages:

Induction Period (0–3 min):

PAC undergoes rapid hydrolysis, releasing Al^3+^ ions that preferentially coordinate with the amine groups of PEI. Simultaneously, an initial hydrogen-bonded network forms between PVA chains. The system’s viscosity increases gradually while maintaining sufficient fluidity, thereby satisfying the requirements for pumpability and fracture penetration.

Rapid Crosslinking Period (3–7 min):

Extensive coordination occurs between Al^3+^ ions and hydroxyl groups on PVA chains, accompanied by the polycondensation of aluminum hydroxo species. These processes trigger the rapid formation of a three-dimensional (3D) interpenetrating PVA–PEI–PAC network. Consequently, the system’s viscosity increases exponentially, while fluidity decreases sharply.

Gel Point (7–8.2 min):

Once the percolation threshold of crosslinking density is reached, the system undergoes a sol–gel transition, forming a self-supporting elastic network that completely loses fluidity. The measured gelation time is 8.2 min. This time window satisfies the dual requirements of mine firefighting engineering: sufficient time for fracture penetration (>5 min) and rapid setting for effective retention (<10 min).

2. Cooperative Roles of Components

PVA acts as the structural backbone. Its hydroxyl groups form a continuous hydrogen-bonded network, providing structural support and active sites for subsequent coordination crosslinking.

PAC serves as the primary regulator of gelation kinetics. Its hydrolysis product, Al^3+^, performs dual functions: (i) forming O → Al coordination bonds with hydroxyl groups of PVA (chemical crosslinking) and (ii) undergoing hydrolysis and polycondensation to generate Al–O–Al inorganic crosslinking nodes. Together, these processes construct an organic–inorganic interpenetrating network.

PEI functions as a network modifier. Its amine groups participate in N → Al coordination and form hydrogen bonds with PVA chains, promoting chain bridging and improving network homogeneity. Although PEI has a limited effect on gelation rate, it significantly enhances thermal stability and structural integrity, preventing network collapse under elevated temperatures.

In summary, the gelation process is cooperatively driven by hydrogen bonding, coordination crosslinking, and inorganic polycondensation. PAC governs the initiation kinetics. PVA determines the network framework, and PEI optimizes structural uniformity. The coupling of these three components enables precise regulation of gelation behavior, resulting in a stable 8.2 min gelation threshold.

### 3.2. Microscopic Structure Characterization Analysis

To analyze the microstructural characteristics of the PVA/PEI/PAC gel and their intrinsic relationship with fire prevention and extinguishing performance, scanning electron microscopy (SEM) was employed to observe the optimal formulation gel and its coal-coated composite system. The results not only visually demonstrate the material morphology but also provide microstructural evidence for its excellent water retention, adhesion, and flame-retardant mechanisms.

**Figure 2:**
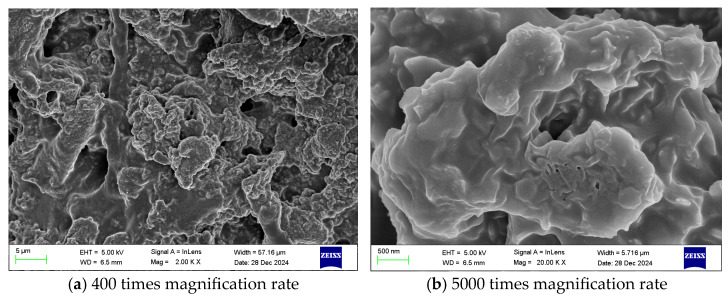
Scanning electron microscope images of the gel at different magnification levels. Reproduced with permission from [35], MDPI, 2026.

Figure 2 shows the microstructure of the pure gel. At low magnification (Figure 2a), the gel exhibits a continuous, defect-free macroscopic morphology, indicating complete reaction and homogeneous formation of all components. At high magnification (Figure 2b), a well-developed three-dimensional network structure is clearly observed. This network consists of a PVA molecular-chain skeleton entangled via hydrogen bonding, which is further stabilized through coordination crosslinking between Al^3+^ ions (derived from PAC) and the hydroxyl groups of PVA as well as the amine groups of PEI. This porous structure serves two key functions: first, its high specific surface area and pore volume enable the physical confinement of a large amount of free water, providing the structural basis for the gel’s excellent water retention capacity; second, the interconnected pore channels facilitate water migration and the diffusion of active ions (e.g., Al^3+^), ensuring their uniform distribution and sustained interaction with the coal surface.

**Figure 3:**
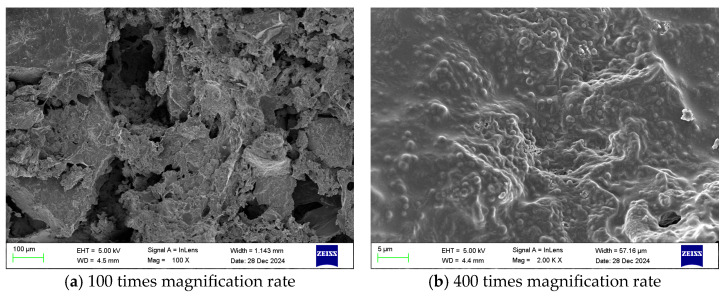
Scanning electron microscope images of the gel + coal sample at different magnification levels. Reproduced with permission from [35], MDPI, 2026.

Figure 3 presents the microscopic morphology of the gel–coal composite system. It is clearly observed that the gel does not merely accumulate around coal particles but instead tightly wraps, infiltrates, and embeds into the surface and fissures of the coal matrix (Figure 3a). At higher magnification (Figure 3b), the interface between the gel and coal becomes indistinct, forming a dense, gap-free composite layer. This phenomenon microscopically confirms the gel’s strong coating and adhesion ability to coal. This intimate contact first creates a physical barrier effect, forming a dense layer on the coal surface that effectively blocks oxygen diffusion. Second, the close interaction significantly increases the probability of contact between the gel’s active functional groups (-OH, -NH_2_, and Al^3+^) and the reactive sites on coal, thereby facilitating the hydrogen bonding, coordination, and condensation interactions identified in the FTIR analysis.

In summary, SEM analysis indicates that the prepared gel successfully constructs a three-dimensional network structure characterized by porosity, interconnectivity, and strong adhesion. This structure is key to understanding its macroscopic performance: the internal interconnected porous framework enables water storage and transport, while the external dense coating layer provides oxygen isolation and strong interfacial bonding. This microstructural design is the fundamental reason why the material can simultaneously achieve multiple fire prevention and extinguishing functions, including oxygen barrier formation, evaporative cooling, and chemical passivation.

### 3.3. Gel Base Performance Test and Result Analysis

#### 3.3.1. Viscosity Testing

Four gel samples with different ratios (10% A + 8% B + 5.5% C, 12% A + 9% B + 5.5% C, 14% A + 7% B + 5.5% C, and 14% A + 8% B + 5% C) were selected for this experiment. The samples were stirred at room temperature, and the gelation process was recorded over time. The changes in gel viscosity within 0–20 min were monitored, and the influence of different component ratios on viscosity was analyzed.

**Figure 4:**
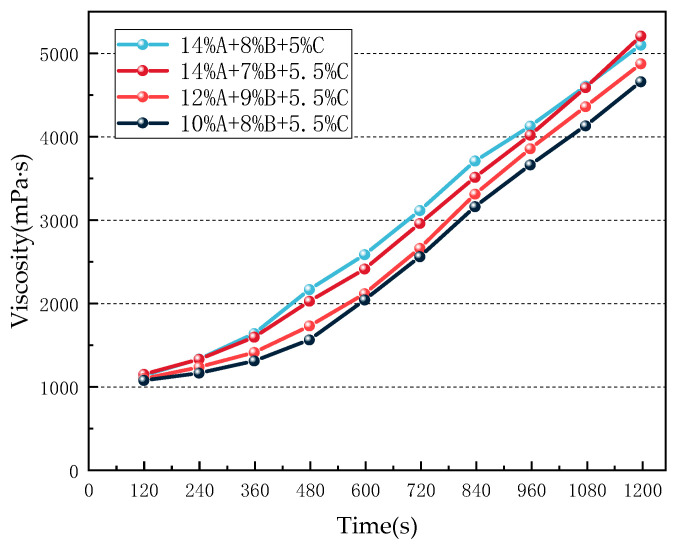
Viscosity change curves of different gel ratios.

The viscosity evolution of gels with different formulations was investigated. The experimental results are shown in Figure 4.

As shown in Figure 4, gel viscosity increased slowly before 360 s, during which the crosslinking reaction gradually proceeded and the release of metal ions was accelerated. After 360 s, the viscosity increased significantly, fluidity decreased, and gelation was eventually completed. The figure also shows that as the concentration of component A increased, gel viscosity increased significantly. This is likely because the increased concentration of A enhanced intermolecular interactions between polymer chains and increased the number of crosslinking sites, resulting in the formation of a more complex and denser network structure, which led to an increase in viscosity.

#### 3.3.2. Strength Test

The settling depth of the glass rod varied significantly at different time points. In the four experiments, the variation in settling depth decreased significantly after 9 min. The settling depth at the 9th minute was approximately 66.91% lower than the average value at 3 min and about 53.66% lower than that at 6 min. This phenomenon indicates that, at 9 min, the gel system became more compact, and the crosslinked chains had gradually formed. From 9 to 15 min, the settling rate decreased further, possibly due to the formation of a stable network structure within the gel, which enhanced gel strength and made it more difficult for the glass rod to settle. Additionally, Table 4 shows that as the concentrations of A and B increased, gel strength increased, indicating that A and B, as the main crosslinking components, form a more stable network structure.

#### 3.3.3. Analysis of Exudation Test

**Figure 5:**
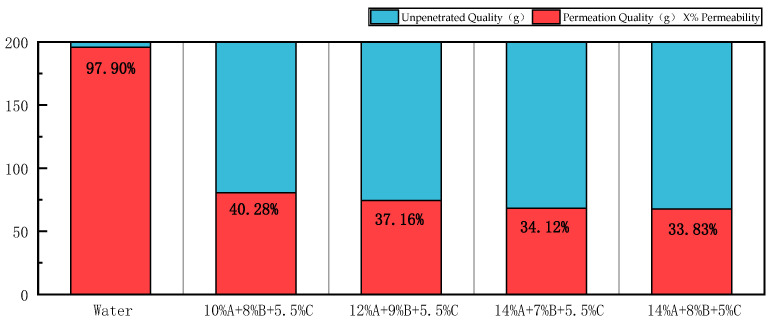
Permeation rate of water and different ratios of gels.

According to the test procedures, water and four groups of the selected gels were tested separately. The amounts of water that permeated into the containers and the masses of the gels were recorded. The permeation rates of water and each group of gels were calculated using the formula. The results are shown in Figure 5.

As can be seen from Figure 5, the water seepage rate is as high as 97.90%, indicating that water hardly remains in coal seam fractures. The average seepage rate of the four gels is 36.35%. This result not only shows that the gels can penetrate into high-temperature zones formed by coal spontaneous combustion but also indicates that part of the gel can remain in coal seam fractures and act as an oxygen barrier. Among them, the gel with the ratio of 10% A + 8% B + 5.5% C exhibits the highest seepage rate, while the gel with the ratio of 14% A + 8% B + 5% C shows the lowest seepage rate. This is because higher concentrations of A and B lead to a more stable internal network structure, higher gel viscosity, and stronger adhesion to coal surfaces and fractures. Therefore, the seepage amount is relatively lower in this case.

### 3.4. Analysis of Gel Fire Extinguishing Performance

#### 3.4.1. Infrared Spectroscopic Analysis

**Figure 6:**
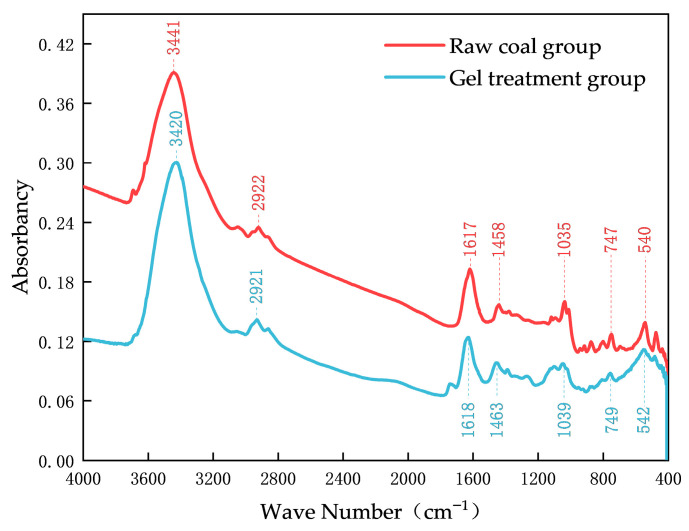
Infrared spectrum graph.

To analyze the characteristic peaks and functional groups of the two sample sets, the experimental curves were smoothed to reduce the influence of noise. The Savitzky–Golay smoothing method was applied, with a smoothing window size of nine and a polynomial order of two. The infrared spectra of the two sample sets are shown in Figure 6.

As shown in Figure 6, the infrared spectra of the two sample sets are generally similar, and characteristic peaks appear in different wavenumber regions. Specifically, obvious peaks or multiple consecutive peaks are observed in the ranges of 3690–3200 cm^−1^, 3110–2800 cm^−1^, 1875–1545 cm^−1^, 1455–1000 cm^−1^, and 900–400 cm^−1^, indicating the presence of functional groups such as hydroxyl groups, hydrogen bonds, methyl groups, methylene groups, and carbonyl groups in the coal samples.

In the gel-treated group, the characteristic absorption peak of hydroxyl groups shifted from 3441 cm^−1^ to 3420 cm^−1^, indicating that components in the gel formed hydrogen bonds or other interactions with hydroxyl groups in coal. This shift reflects a change in the vibrational frequency of the hydroxyl groups and suggests enhanced interaction between the gel and coal, thereby improving the adhesion and stability of the gel system.

To analyze the distribution characteristics of active functional groups in the two sample sets, the infrared spectra were divided into six wavenumber intervals based on the spectral peak assignment table of functional groups in coal molecules: 3690–3200 cm^−1^, 3100–2800 cm^−1^, 1710–1150 cm^−1^, 1150–1000 cm^−1^, 960–675 cm^−1^, and 675–400 cm^−1^. The infrared absorption spectra of the two sample sets were processed using peak fitting in Origin software (2020). Second-derivative processing was applied to each wavenumber interval, and the number of sub-peaks was determined according to the number of minima in the second-derivative curves. Gaussian functions were then used for curve fitting. To ensure complete convergence of the fitting results, the fitting parameters were repeatedly adjusted and optimized using the least-squares method.

**Figure 7:**
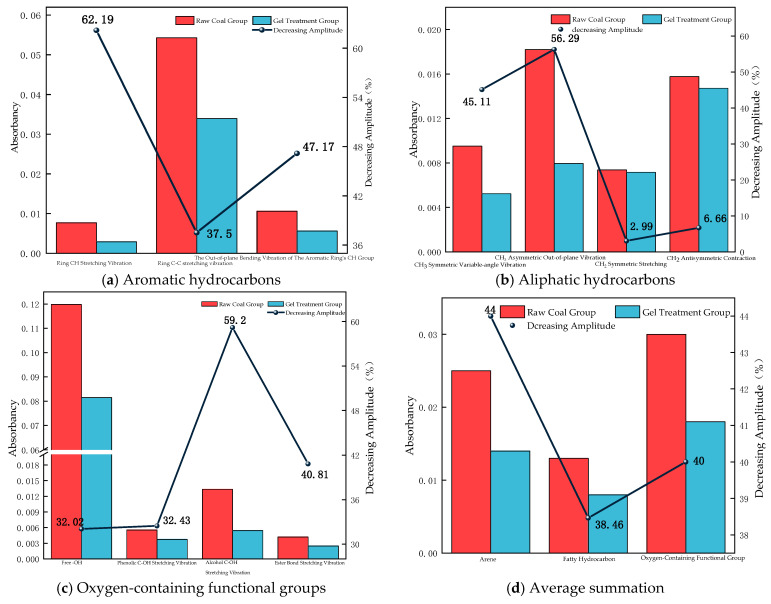
Changes in absorbance and their reductions.

**Figure 8:**
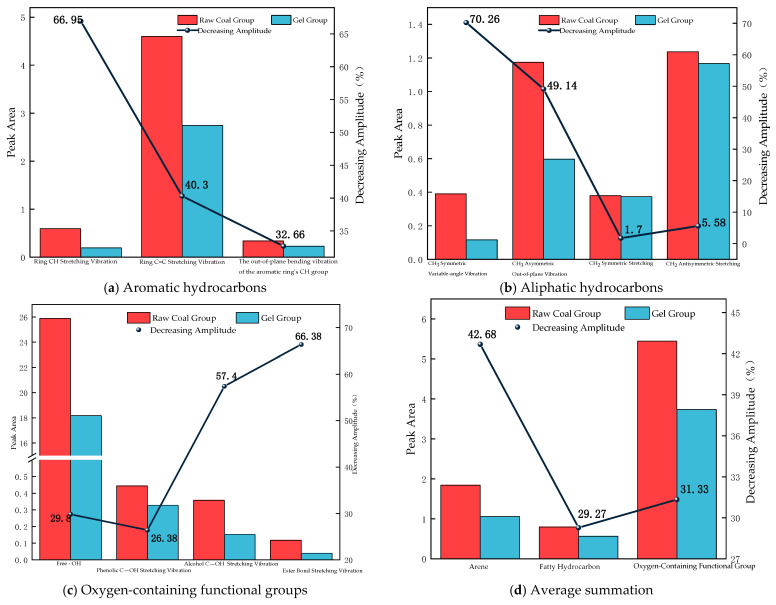
Peak area changes and their decline rates.

Peak-fitting analysis of the infrared spectra was subsequently performed. The peak area and absorbance values can reflect changes in the active functional groups in the samples. Based on the peak-fitting results, the average values of the functional group contents in the raw coal group and the gel-treated group were calculated to evaluate the influence of the gel on functional group distribution in raw coal. The variations in absorbance (Figure 7) and peak area (Figure 8) are presented using a dual Y-axis plot.

As shown in Figure 7d, the absorbance of aromatic hydrocarbons in the raw coal group is 0.025, while that in the gel-treated group is 0.014. Compared with the raw coal group, the absorbance decreases by 44%. As shown in Figure 7a, the gel significantly reduces the intensity of the C–H stretching vibration of the aromatic ring, the C=C stretching vibration of the aromatic ring, and the out-of-plane C–H bending vibration of the aromatic ring.

The absorbance of aliphatic hydrocarbons in the raw coal group is 0.013, while that in the gel-treated group decreases to 0.008, representing a 38.46% reduction compared with the raw coal group. As shown in Figure 7b, the results indicate that the gel significantly reduces the intensity of the symmetric and asymmetric bending vibrations of CH_3_ and slightly reduces the intensity of the symmetric and asymmetric stretching vibrations of CH_2_.

In addition, the absorbance of oxygen-containing functional groups in the raw coal group is 0.03, while that in the gel-treated group decreases to 0.018, indicating a 40% reduction compared with the raw coal group. As shown in Figure 7c, the intensities of the stretching vibrations of free –OH, phenolic C–OH, aliphatic C–OH, and ether bonds all decrease. Among them, the absorbance of free –OH in the raw coal sample is 0.11984, which decreases to 0.08147 in the gel-treated group, reflecting a 32.02% reduction and indicating that the gel has a pronounced inhibitory effect on free –OH groups.

As shown in Figure 8d, the peak area of aromatic hydrocarbons in the raw coal group is 1.844, while that in the gel-treated group decreases to 1.057, representing a reduction of 42.68%. As shown in Figure 8a, the gel significantly reduces the intensity of the C–H stretching vibration of the aromatic ring, the C=C stretching vibration of the aromatic ring, and the out-of-plane C–H bending vibration of the aromatic ring. Among these, the reduction in the C–H stretching vibration is particularly significant, reaching 66.95%.

The peak area of aliphatic hydrocarbons in the raw coal group is 0.796, which decreases to 0.563 in the gel-treated group, reflecting a reduction of 29.27%. As shown in Figure 8b, the results indicate that the gel significantly reduces the intensity of the symmetric and asymmetric bending vibrations of CH_3_ and the symmetric and asymmetric stretching vibrations of CH_2_, indicating that the gel has a pronounced inhibitory effect on CH_3_-related vibrations.

In addition, the peak area of the oxygen-containing functional groups in the raw coal group is 5.442, decreasing to 3.737 in the gel-treated group, which reflects a reduction of 31.33%. As shown in Figure 8c, the intensities of the stretching vibrations of free –OH, phenolic C–OH, alcoholic C–OH, and ether bonds all decrease. Among the oxygen-containing functional groups, the peak area of free –OH in the raw coal group is 25.881, which decreases to 18.167 after gel treatment, representing the largest reduction (29.8%) and further confirming that the gel has a strong inhibitory effect on free –OH groups.

Based on the existing FTIR data, analysis of characteristic peak shifts and peak area changes allows distinction between two types of chemical interactions: internal crosslinking within the gel and passivation at the gel–coal interface.

Internal Crosslinking Mechanism of the Gel (Revealed by Peak Shifts):

The O–H stretching vibration peak of PVA red-shifts from 3441 cm^−1^ to 3420 cm^−1^, indicating the formation of a hydrogen-bonded network with the –NH_2_ groups of PEI. The shifts in the amine deformation vibration peak of PEI (~1640 cm^−1^) and the C–OH vibration peak of PVA (~1080 cm^−1^) indicate the formation of N/O → Al coordination bonds between Al^3+^ and N/O atoms. The broadened peak in the 700–500 cm^−1^ region corresponds to the formation of –Al–O–Al– inorganic network nodes. These shifts suggest that the internal three-dimensional network is cooperatively constructed through hydrogen bonding, coordination bonding, and inorganic polycondensation.

Gel–Coal Interface Passivation Mechanism (Revealed by Peak Area Reduction):

The peak areas of active functional groups on the coal surface show a significant reduction. Free –OH groups decrease by 29.8%, while aromatic hydrocarbons, aliphatic hydrocarbons, and oxygen-containing functional groups decrease by 42.68%, 29.27%, and 31.33%, respectively. This indicates that the gel components are chemically anchored to the coal surface via coordination, hydrogen bonding, and other interactions, rather than through mere physical coverage, thereby effectively passivating oxidation-active sites on the coal.

In summary, the FTIR results clearly indicate that internal crosslinking (peak shifts) constructs a stable gel network, while interface passivation (peak area reduction) provides long-term chemical protection for coal. The cooperative action of these two processes forms the microscopic basis of the material’s efficient fire prevention and extinguishing performance.

#### 3.4.2. TG-DSC Analysis

**Figure 9:**
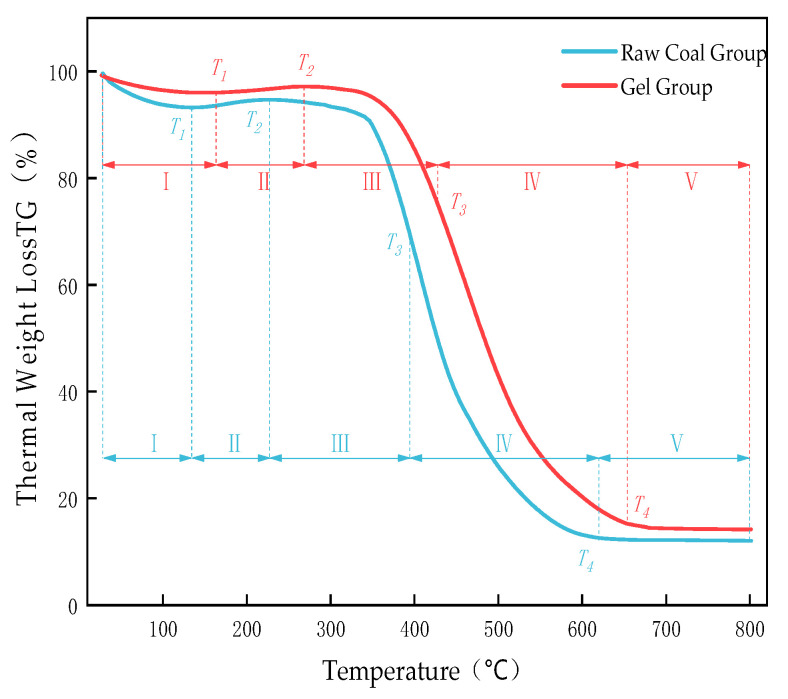
Sample TG curve.

The TG curves of the raw coal group and the gel-treated group are shown in Figure 9, and the characteristic temperatures are presented in Table 5.

**Figure 10:**
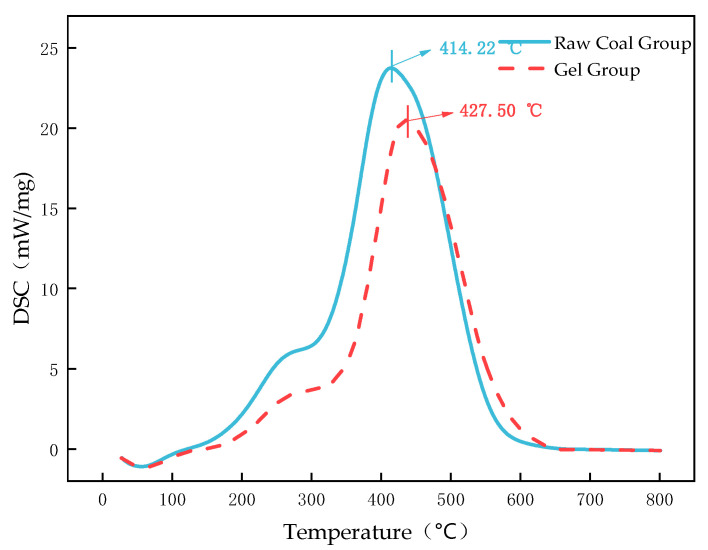
DSC curve of the sample.

Combined analysis of thermogravimetric (TG) and differential scanning calorimetry (DSC) curves allows the deconstruction of the programmed coal temperature oxidation process into five consecutive stages. Each stage shows a one-to-one correspondence between mass change (TG) and thermal effects (DSC). The DSC curve of the sample is shown in the Figure 10.

Stage I (Room Temperature~T_1_): Dehydration and Desorption (Endothermic)

In this stage, the TG curve shows a slow decline, corresponding to the removal of physically adsorbed water and some light hydrocarbons. The DSC curve in this interval exhibits a broad, shallow endothermic trough, representing the heat absorption associated with dehydration and desorption. For the gel-treated coal, T_1_ is significantly delayed from 134.21 °C to 163.64 °C, and the TG curve remains at a higher level, providing evidence of the gel’s excellent moisture retention capability, which delays the initial drying of coal.

Stage II (T_1_~T_2_): Oxygen Uptake and Weight Gain (Weakly Exothermic)

Active sites in coal undergo low-temperature oxidation reactions with oxygen, generating unstable oxygen-containing complexes and leading to a slight weight increase in the TG curve. This process is typically accompanied by slow heat release, which is manifested as a slight upward baseline shift (weak exotherm) in the DSC curve. The T_2_ of the gel-treated coal is significantly delayed from 227.37 °C to 268.35 °C, indicating that the gel effectively inhibits oxygen diffusion to the coal surface, thereby delaying the low-temperature oxidation process.

Stage III (T_2_~T_3_): Thermal Decomposition and Volatile Release (Predominantly Endothermic)

Unstable oxygen-containing complexes decompose, and weak bonds such as aliphatic side chains in coal break, producing CO, CO_2_, and light hydrocarbon volatiles. This causes the TG curve to begin decreasing at an accelerating rate. As this process is dominated by bond cleavage (endothermic), it corresponds to an endothermic background or plateau preceding the main exothermic peak in the DSC curve. The ignition temperature (T_3_) of the gel-treated coal increases from 394.23 °C to 427.50 °C, demonstrating that the gel and its reaction products enhance the thermal stability of coal and increase the temperature threshold for the onset of intense combustion.

Stage IV (T_3_~Burnout): Intense Combustion (Strongly Exothermic)

This is the main combustion stage, where fixed carbon and remaining volatiles react violently with oxygen. The TG curve shows a sharp decrease, and the DTG curve exhibits a peak (T_4_). This rapid mass loss corresponds to the main exothermic peak in the DSC curve. For raw coal, the main exothermic peak temperature (T_p_) is 414.22 °C, coinciding with the temperature of the maximum mass loss rate (T_4_). For gel-treated coal, this peak temperature is delayed to 427.50 °C, and the peak area is significantly reduced. This indicates that the gel not only delays the intense combustion stage by 13.28 °C but also substantially reduces the total heat release during this stage. This is attributed to the cooperative effects of chemical passivation of active coal functional groups (see FTIR analysis) and physical oxygen barrier formation.

Stage V (Post-Burnout): Ash Formation

The TG curve levels off, leaving residual ash. The final residue mass of the gel-treated coal is slightly higher, possibly due to the presence of inorganic components from the gel (e.g., Al_2_O_3_).

Combined TG–DSC analysis clearly demonstrates that the inhibitory effect of the gel spans the entire spontaneous coal combustion process. In the low-temperature stages (I–III), it mainly delays oxygen uptake and pyrolysis through moisture retention, oxygen isolation, and thermal stabilization. In the high-temperature combustion stage (IV), it significantly increases the ignition temperature, delays the main combustion reaction, and reduces the total heat release through chemical passivation and continuous coverage.

The strong correspondence and synchronous delay between the main exothermic peak in DSC (heat release) and the maximum mass-loss rate in TG (fixed carbon combustion) provide thermodynamic evidence of the gel’s high-efficiency flame-retardant performance.

#### 3.4.3. Fire Extinguishing Performance Test

Test Group 1: Prepare 5% (100 g) clear water and set it aside.

Test Group 2: Prepare a 5% (100 g) sodium silicate gel and set it aside.

Test Group 3: Prepare a 5% (100 g) gel with a ratio of 14% A + 7% B + 5.5% C and set it aside.

Test Group 4: Prepare a 10% (200 g) gel with a ratio of 14% A + 7% B + 5.5% C and set it aside.

**Figure 11:**
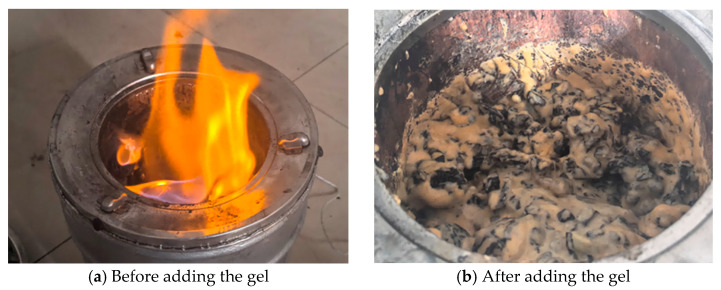
Comparison before and after gel fire extinguishing.

**Figure 12:**
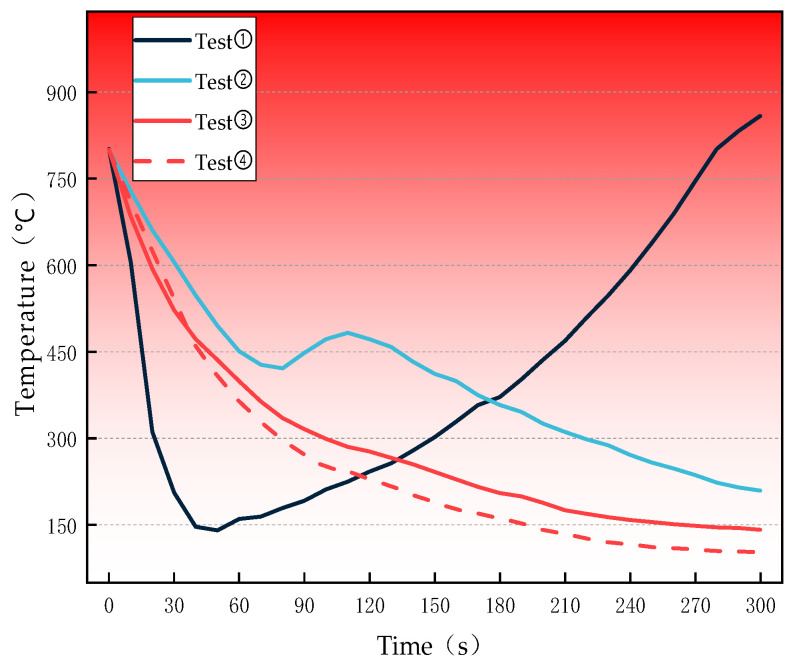
Temperature curve chart of the fire-extinguishing experiment.

The fire-extinguishing performance of the gel before and after application is presented in Figure 11, and the corresponding temperature variations during the fire-extinguishing process are illustrated in Figure 12.

Due to the high fluidity of water, the temperature in Test Group 1 decreased rapidly in the initial stage. However, as the water evaporated, the coal temperature dropped to approximately 140 °C and then reignited. Test Group 2 also experienced reignition when the coal temperature decreased to 420 °C. This is likely because the silica gel lost water and cracked at high temperatures, reducing its coverage and oxygen isolation effect on the coal and resulting in air leakage and subsequent reignition.

In Test Groups 3 and 4, after the addition of the gel with a ratio of 14% A + 7% B + 5.5% C, the coal temperature decreased rapidly, and the cooling rate gradually slowed down. Throughout the entire process, no reignition occurred, and the cooling effect was significantly better than that of the conventional silica gel. Moreover, the 10% gel exhibited a better fire-extinguishing performance than the 5% gel.

#### 3.4.4. Fire-Extinguishing Principle of the Gel

The results of the comprehensive fire-extinguishing experiments and the tests on the basic properties, microstructure, and flame retardancy of the gel indicate that this gel material achieves its fire prevention and extinguishing effect mainly through chemical passivation. At the microscopic level, the hydroxyl groups (–OH) in the gel form hydrogen bonds with the active –OH groups in coal, and aluminum ions (Al^3+^) coordinate with free –OH groups. In addition, H^+^ ions are released, promoting condensation reactions between amino groups (–NH_2_) in the gel and active groups in coal, thereby effectively reducing the reactivity of active functional groups in coal and inhibiting the coal oxidation process.

PVA−O−H•••O−H−R

$$\mathrm{PAC}-{\mathrm{Al}}^{3+}+R-\mathrm{OH}\to R-O-{\mathrm{Al}}^{2+}+H^{+}$$

$$\mathrm{PEI}-{\mathrm{NH}}_{2}+R-\mathrm{OH}\overset{H^{+}}{\to }R-\mathrm{NH}-{\mathrm{PEI}+H}_{2}O$$

Since 70% of the gel composition consists of water, it can effectively provide wetting and evaporative cooling when applied to coal, thereby absorbing heat. However, due to the strong fluidity of water, it cannot remain effective on the coal surface for a long period. In contrast, the gel exhibits excellent water retention properties. Its internal porous structure can effectively retain moisture, thereby prolonging water evaporation and heat absorption and ensuring a more sustained fire-extinguishing effect.

**Figure 13:**
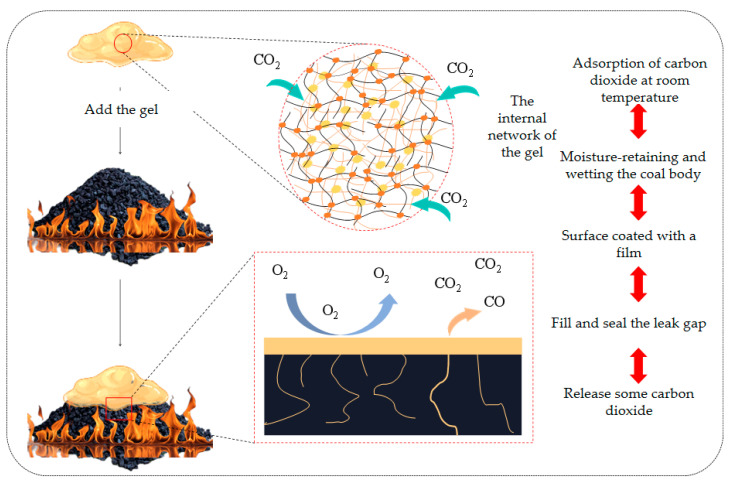
Mechanism diagram of the fire-extinguishing gel.

When the gel agent is injected, it forms a continuous coating layer on the coal surface. As water gradually evaporates, the gel transforms into a membrane-like structure. This effectively isolates the coal from external oxygen and slows down the oxidation rate. At the same time, part of the gel penetrates into coal fractures and adheres to the interior of the coal through adhesion, filling voids within the coal and blocking air leakage channels. This further reduces the contact area between coal and oxygen, thereby more effectively inhibiting oxygen diffusion. The fire-extinguishing mechanism of the gel is shown in Figure 13.

#### 3.4.5. Analysis of the Cooperative Fire Prevention and Extinguishing Mechanism of the Ternary Gel

1. Chemical Passivation at the Gel–Coal Interface

The gel not only covers the coal surface but also actively passivates its reactive sites through chemical reactions, a mechanism confirmed by FTIR analysis.

Physical Encapsulation and Oxygen Barrier: SEM confirms that the gel forms a dense, continuous coating on the coal surface (Figure 3), which first physically blocks oxygen diffusion, thereby delaying low-temperature oxidation of coal, as evidenced by the delayed weight-gain stage in TG analysis.

Multifaceted Chemical Passivation: The active components in the gel undergo specific reactions with functional groups on the coal surface:

Hydrogen Bonding (PVA–O–H···O–H–R (coal)): This interaction causes a red shift in the O–H stretching vibration peak in the FTIR spectrum (from 3441 to 3420 cm^−1^), enhancing gel adhesion to the coal surface.

Coordination and Proton Release (PAC–Al^3+^ + R–OH (coal) → R–O–Al^2+^ + H^+^): Al^3+^ coordinates with hydroxyl groups in coal, fixing active sites while simultaneously releasing H^+^.

Condensation Reaction Catalyzed by H^+^, PEI–NH_2_ + R–OH (coal) → R–NH–PEI + H_2_O: This reaction consumes key free hydroxyl groups in coal.

These reactions collectively lead to a significant reduction in the FTIR peak areas of aliphatic hydrocarbons, aromatic hydrocarbons, and oxygen-containing functional groups (especially free –OH, with a reduction of up to 29.8%) in coal, thereby lowering its chemical reactivity.

2. Cooperative Fire Prevention and Extinguishing by the Ternary Gel

Ultimately, the gel achieves highly efficient fire prevention and suppression through the following mechanisms:

Initial Stage (Injection and Penetration): The controllable gelation time (8.2 min) ensures that the precursor solution fully penetrates coal fractures before rapidly crosslinking, setting, and forming a stable retained coating.

Mid-Term Stage (Cooling and Oxygen Isolation): Water retained in the gel evaporates endothermically, providing significant cooling. The formed elastic gel layer and the subsequent solid film after drying provide continuous physical oxygen isolation.

Long-Term Stage (Anti-Reignition and Inhibition): The thermally stable network enhanced by PEI prevents cracking at high temperatures. The sustained release of Al^3+^ and chemical passivation products provides long-term inhibition of coal, fundamentally disrupting the combustion chain reaction.

The delayed ignition point (ΔT = 33.27 °C), postponed maximum exothermic peak temperature (ΔT = 13.28 °C), and reduced total heat release observed in TG–DSC data are direct manifestations of these physicochemical cooperative effects.

## 4. Conclusions

A multifunctional fire prevention and extinguishing gel was prepared and investigated to determine its performance and coal spontaneous combustion suppression characteristics. The following conclusions were drawn:

(1) Through a three-factor, three-level orthogonal experiment and variance analysis (R^2^ = 0.987), the significance of each factor on gelation time followed the order of PAC (F = 49.000, *p* = 0.020) > PVA (F = 23.258, *p* = 0.041) > PEI (*p* = 0.189). The optimal formulation was 14% PVA + 7% PEI + 5.5% PAC (mass fraction), with a gelation time of 8.2 min, meeting the requirements for fracture penetration in coal mines.

(2) The gel formed a dense, interconnected three-dimensional network structure at the microscopic level, which closely covered the coal surface and filled fractures. At the macroscopic level, it exhibited ideal viscosity evolution and mechanical strength. At 9 min, the penetration depth of the glass rod was 12.4 mm and stabilized at 11.5 mm at 15 min. The average leakage rate was only 36.35%, which is much lower than that of water (97.90%), indicating a significant retention and protective effect.

(3) FTIR analysis showed that the peak area of free hydroxyl groups in the coal sample decreased by 29.8%, while the peak areas of aromatic hydrocarbons, aliphatic hydrocarbons, and oxygen-containing functional groups decreased by 42.68%, 29.27%, and 31.33%, respectively, effectively deactivating reactive functional groups. TG–DSC results indicated that the ignition temperature of the coal sample was delayed by 33.27 °C, the maximum exothermic peak temperature was delayed by 13.28 °C, the burnout temperature was delayed by 34.05 °C, and thermal stability was significantly improved.

(4) Fire-extinguishing experiments confirmed that the gel could continuously cool an 800 °C coal sample without reignition, significantly outperforming water (reignition at 140 °C) and conventional sodium silicate gel (reignition at 420 °C). The 10% gel exhibited better performance than the 5% gel, and its effectiveness was attributed to the cooperative effects of water-retentive cooling, physical oxygen isolation, and chemical inactivation.

(5) The results indicate distinct cooperative roles of the components: PVA provides structural support. PAC controls gelation kinetics, and PEI enhances structural stability. The constructed cooperative “network–interface passivation” system enables integrated fire prevention performance.

## Figures and Tables

**Table 1:** Orthogonal experimental design [35].

Experiment Number		Factor	
	A PVA Content (%)	B PEI Content (%)	C PAC Content (%)
1	10.0	7.0	4.5
2	10.0	8.0	5.5
3	10.0	9.0	5.0
4	12.0	7.0	5.0
5	12.0	8.0	4.5
6	12.0	9.0	5.5
7	14.0	7.0	5.5
8	14.0	8.0	5.0
9	14.0	9.0	4.5

**Table 2:** Results of the orthogonal experiment [35].

Experiment Number	PVA Content (%)	PEI Content (%)	PAC Content (%)	Gelation Time (min)
1	10.0	7.0	4.5	20.40
2	10.0	8.0	5.5	9.95
3	10.0	9.0	5.0	15.55
4	12.0	7.0	5.0	13.45
5	12.0	8.0	4.5	16.15
6	12.0	9.0	5.5	9.00
7	14.0	7.0	5.5	8.20
8	14.0	8.0	5.0	9.35
9	14.0	9.0	4.5	13.25

**Table 3:** Significance analysis of the effects of CS, AM, and MBA contents on gel formation time [35].

Source of Difference	Sum of Squares of Deviations	Degree of Freedom	Mean Square	F	*p*-Value
PVA	40.056	2	20.028	23.258	0.041
PEI	7.389	2	3.694	4.290	0.189
PAC	84.389	2	42.194	49.000	0.020

*p* < 0.05 (significant).

**Table 4:** Gel strength test.

Grouping	Time (min)	Depth (mm)
10% A + 8% B + 5.5% C	3	41.1
	6	30.2
	9	14.1
	12	12.8
	15	12.1
12% A + 9% B + 5.5% C	3	40.5
	6	28.5
	9	13.7
	12	12.5
	15	11.9
14% A + 7% B + 5.5% C	3	38.5
	6	27.6
	9	12.4
	12	11.8
	15	11.5
14% A + 8% B + 5% C	3	37.8
	6	26.5
	9	12.1
	12	11.5
	15	10.9

**Table 5:** Sample characteristic point temperatures.

Specimen	Initial Minimum Weight Temperature T1 (°C)	Maximum Weight Temperature T2 (°C)	Ignition Temperature T3 (°C)	Burnout Temperature T4 (°C)
Raw Coal Group	134.21	227.37	394.23	619.53
Gel Treatment Group	163.64	268.35	427.50	653.58

## Data Availability

Date are contained within the article.
